# *Radix Sophorae Flavescentis* induces apoptosis through by Caspase, MAPK Activation and ROS Signaling Pathways in 5637 Human Bladder Cancer Cells

**DOI:** 10.7150/ijms.45831

**Published:** 2020-06-15

**Authors:** Geoncheol Jo, Min Ji Kwon, Jeong Nam Kim, Byung Joo Kim

**Affiliations:** Division of Longevity and Biofunctional Medicine, Pusan National University School of Korean Medicine, Yangsan 50612, Republic of Korea.

**Keywords:** *Radix Sophorae Flavescentis*, Apoptosis, Proliferation, Bladder cancer, 5637

## Abstract

The anti-cancer mechanisms of *Radix Sophorae Flavescentis* were investigated in 5637 bladder cancer cells. *Radix Sophorae Flavescentis* extract (RSF) (50‑400 µg/ml) inhibited the proliferation of 5637 cells and increased sub‑G1 phase ratios. RSF‑induced cell death was associated with the down-regulation of B‑cell lymphoma 2 (Bcl‑2) and the up-regulation of Bcl‑2 X‑associated protein (Bax). RSF also activated caspase‑3 and -9 and regulated the activations of mitogen-activated protein kinases (MAPKs). In addition, RSF increased intracellular reactive oxygen species (ROS) levels and depolarized the mitochondrial membrane potential. These findings suggest RSF induces apoptosis in 5637 bladder cancer cells and that it has potential use as a novel anti-cancer drug for bladder cancer.

## Introduction

Bladder cancer is one of the most common cancers in the world in the top 10 and is diagnosed with approximately 550,000 new cancers each year 1. Also, it is difficult to treat because it easily metastasizes and reoccurs 2. Until now, chemotherapy has been viewed as the most effective way of treating bladder cancer 3. However, chemotherapy damages normal tissues and has serious side-effects in bladder cancer patients. Therefore, novel anti-cancer drugs with limited effects on normal cells are required 4,5.

Traditional medicine is widely used to treat cancer because it has fewer complications and side effects and is higher quality of life 6,7. *Radix Sophorae Flavescentis* is an herb that is used in traditional Chinese medicine to treat and prevent various diseases 8. In particular, this shrub has anti-cancer effects, for example, it has been reported to induce the apoptosis of laryngeal cancer Hep2 cells 9, esophageal cancer TE‑8 cells 10, PC‑3 prostate cancer cells 11, and gastric cancer cells 12.

Apoptosis provides an important key to cancer treatment strategies, and the signaling pathway associated with cancer cell growth is viewed as a major target for cancer treatment 13,14. However, relatively little is known about the anti-cancer efficacy of *Radix Sophorae Flavescentis* and even less is known of the mechanisms responsible for its effects. In the present study, we investigated the anti-cancer mechanisms of an ethanolic extract of *Radix Sophorae Flavescentis* in 5637 bladder cancer cells.

## Material and Methods

### Cell culture

Three human urinary bladder cancer cell lines (5637, RT4 and T24) were used. Among them, we used mainly 5637 cell line. Cells were propagated in RPMI-1640 medium (Gibco-BRL, St. Louis, MO, USA) supplemented with 10% heat-inactivated fetal bovine serum (Invitrogen, Grand Island, NY, USA) containing 1% penicillin/streptomycin (Invitrogen, Grand Island, NY, USA) at 37°C.

### Preparation of RSF

RSF extract (RSF) was obtained using ethyl alcohol as described previously 12. Matrine and oxymatrine have long been regarded as major components of the pharmacological efficacy of RSF 15,16. Matrine and oxymatrine were detected by high‑performance liquid chromatography (HPLC) at retention times of 11.6 and 25.7 min, respectively 12. The concentrations of matrine and oxymatrine in RSF were 70.47 ± 1.27 and 299.87 ± 3.746 mg/g, respectively.

### MTT (3-[4,5-dimethylthiazol-2-yl]-2,5-diphenyltetrazolium bromide) assay

5637 cells were seeded onto 12‐well plates at a density of 3 × 10^4^ cells/well. Cell viabilities were determined using an MTT assay (Sigma-Aldrich, St. Louis, MO, USA). 5637 cells were treated with MTT solution and incubated for 2 h at 37°C, following which, absorbance was measured at 570 nm.

### Measurement of cell cycle

5637 cells were treated with ethyl alcohol and vortexed prior to overnight incubation at 4°C. Samples were centrifuged for 5 min and the supernatant was discarded. Cell pellets were resuspended in propidium iodine (PI) staining solution (PI [5 mg/ml; 2 μl) containing RNase (2 μl), spun at 20000 *g* for 10 s, and incubated for 40 min in the dark at room temperature. Samples were analyzed using a fluorescence-activated cell sorter (FACScan; Becton-Dickinson, Mountain View, CA, USA) at λ=488 nm using Cell-Quest software (Becton-Dickinson, Franklin Lakes, NJ, USA).

### Measurement of mitochondrial depolarization assay

For mitochondrial depolarization assay, the cells were treated with 50 nM tetramethylrhodamine methyl ester (TMRM; Sigma-Aldrich, St. Louis, MO, USA) for 30 min. Fluorescence intensities of these samples were measured using a BD FACSCANTO II (BD Biosciences, Sunnyvale, CA, USA) at the excitation and emission wavelengths of 510 and 580 nm, respectively.

### Western blot analysis

Lysates were prepared by incubating cells in RIPA buffer containing protease and phosphatase inhibitor cocktail (Calbiochem, La Jolla, CA, USA). The total protein extracted from the cells was quantified using the Bradford method (Bio‑Rad Laboratories, Hercules, CA). An equal amount of protein (20 μg per lane) from the samples was separated by 8% or 10% SDS-PAGE and probed with indicated antibodies. Antibodies against survivin (#2808), ERK (#9102), pERK (#9106), JNK (#9252), pJNK (#9251), p38 (#9212), and pp38 (#9216) were purchased from Cell Signaling Technology (Danvers, MA, USA), and antibodies against BCl (#sc-783), Bax (#sc-493), caspase-3 (#sc-7148), caspase-9 (#sc-7885), PARP (#sc-7150), β-actin (#sc-47778) and GAPDH (#sc-32233) were from Santa Cruz Biotechnology (Santa Cruz, CA, USA). The secondary horseradish peroxidase‑conjugated antibodies used were goat anti‑rabbit IgG and goat anti‑mouse IgG (cat. nos. SC‑2004 and SC-2005, respectively; Santa Cruz Biotechnology, Dallas, TX, USA). Relative intensities of protein bands were analyzed with a GS‑710 Image Densitometer (Bio‑Rad Laboratories, Hercules, CA, USA). Results are representative of at least five independent experiments.

### Caspase assay

Assays were performed using caspase-3 and -9 assay kits (Cellular Activity Assay Kit Plus; BioMol, Plymouth, PA, USA). After resuspending the cells in ice‑cold cell lysis buffer, the supernatant was removed. Supernatant samples were incubated with caspase substrate (400‑lM Ac‑DEVD‑pNA; 50 μl) at 37°C. Each sample was read at 405 nm at several time‑points.

### Measurement of ROS levels

ROS generation was measured using DCF-DA (2',7'-dichlorodihydrofluorescein diacetate; Molecular Probes, Eugene, OR, USA). The cells were treated with 20 μl DCF‑DA at 37˚C for 30 min and washed with PBS. Fluorescence was measured using FACS (Becton-Dickinson, Mountain View, CA, USA), at excitation/emission wavelengths of 488/525 nm, respectively.

### Statistical analyses

One-way ANOVA with Tukey's *post hoc* comparison was used for multiple comparisons. The analysis was performed using Prism 6.0 (GraphPad Software Inc., La Jolla, CA, USA) and Origin 8.0 (OriginLab Corporation, Northampton, MA, USA) software. Results are expressed as means ± SEMs, and *P* values of < 0.05 were considered statistically significant.

## Results

### Apoptotic effects of RSF in bladder cancer cells

**Figure 1:**
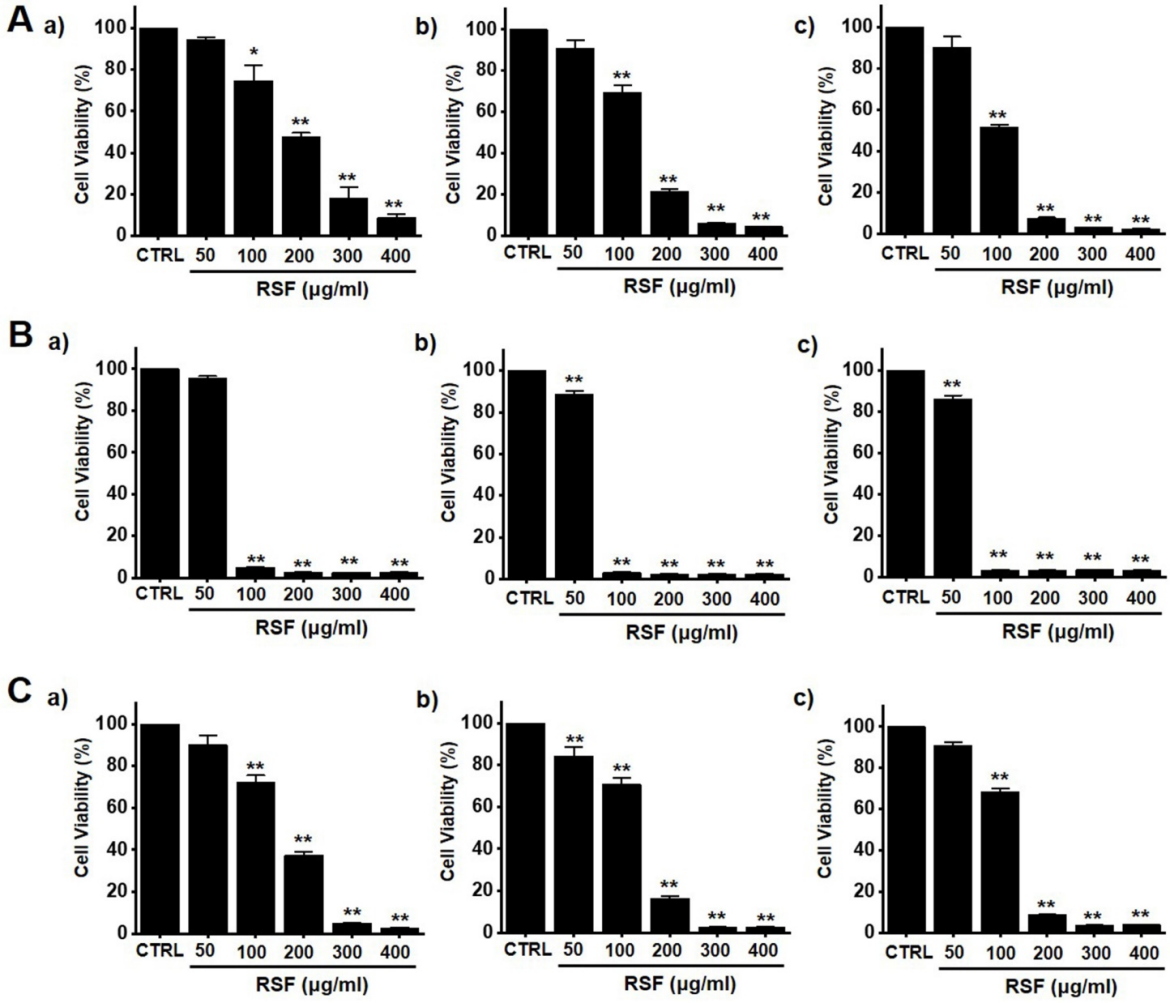
RSF reduced bladder cancer cell viability. Cell viabilities were determined using an MTT assay. (A) RSF reduced cell viabilities dose-dependently in 5637 cells. (B) RSF reduced cell viabilities dose-dependently in RT4 cells. (C) RSF reduced cell viabilities dose-dependently in T24 cells. Results are presented as means ± SEMs. *p<0.05. **p<0.01. RSF, ethanolic extract of *Radix Sophorae Flavescentis*; CTRL, control.

**Figure 2:**
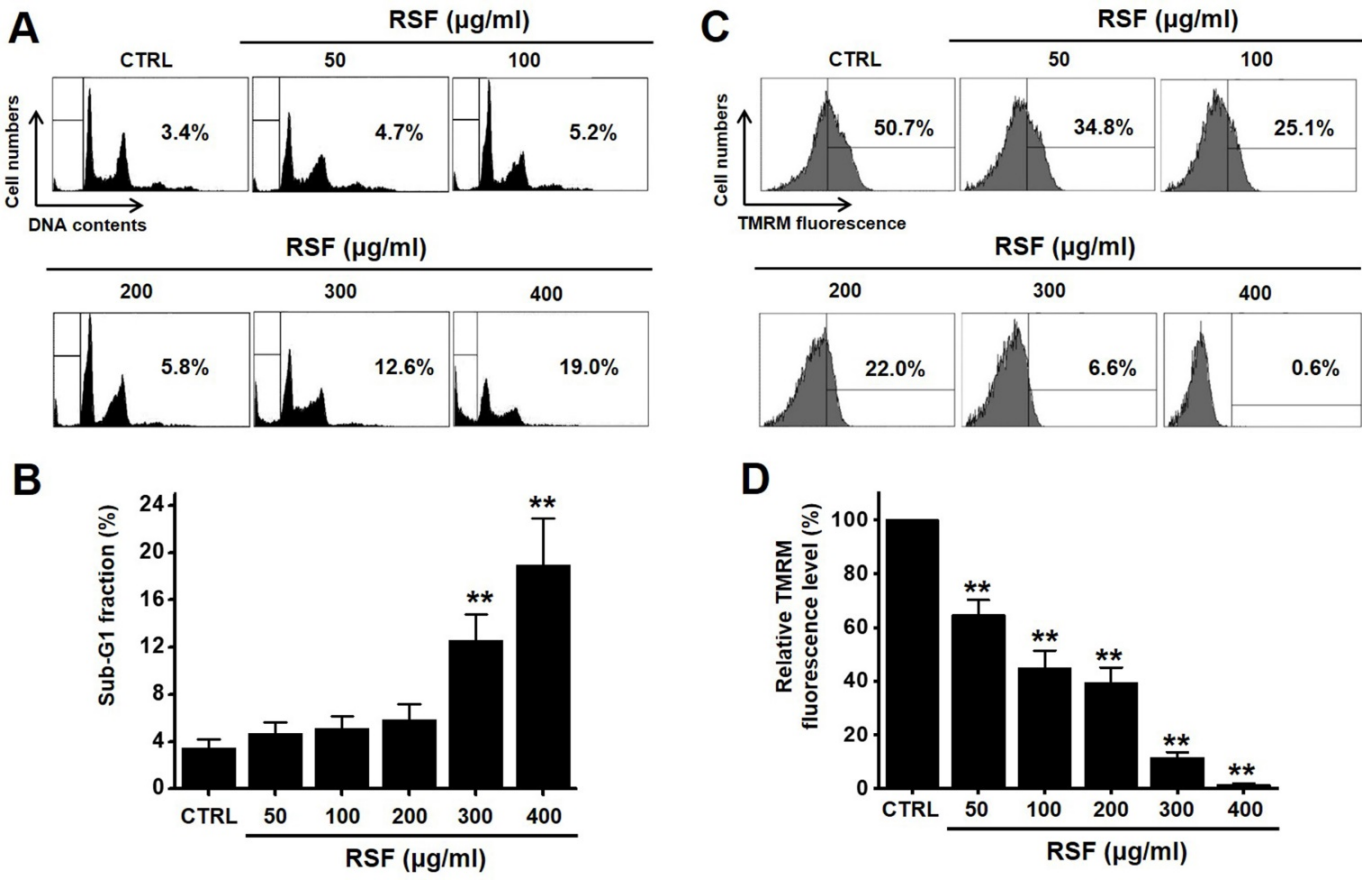
RSF induced sub‑G1 phase ratio increase and mitochondrial damage. (A) Cell cycle analysis was conducted by flow cytometry. (B) Sub-G1 fractions are expressed as percentages. (C) The fluorescence for mitochondrial depolarization assay was measured by FACS analysis. (D) The relative mitochondrial TMRM fluorescence levels were calculated to determine the fold difference in comparison to control. Results are presented as means ± SEMs. *p<0.05. **p<0.01. RSF, ethanolic extract of *Radix Sophorae Flavescentis*; CTRL, control.

To determine whether RSF suppresses bladder cancer cell (5637, RT4 and T24) growth, MTT assays were performed after treating cells with RSF for 24 h, 47 h and 72 h. RSF (50, 100, 200, 300, or 400 µg/ml) reduced the survival of 5637, RT4 and T24 cells by concentration and time dependence (Fig. 1A-1C). Among them, various experiments were conducted mainly with 5637 cells. 5637 cells are in the advanced stage of human bladder cancer, with high capacity of proliferation and metastasis 17. In addition, cell cycle analysis was performed using flow cytometry to determine whether RSF induced apoptosis. Sub-G1 phase ratios were increased by RSF by 4.7 ± 1.0 % at 50 µg/ml, 5.2 ± 0.9 % at 100 µg/ml, 5.8 ± 1.4 % at 200 µg/ml, 12.6 ± 2.3 % at 300 µg/ml, and 18.9 ± 4.0 % at 400 µg/ml as compared with non-treated treated cells by (Fig. 2A and 2B). Also, the mitochondrial depolarization was examined by TMRM staining and the results showed that the mitochondrial membrane of the cells was significantly depolarized on RSF treatment (Fig. 2C and 2D). These results indicate RSF inhibits the proliferation of 5637 cells and that these effects are associated with the induction of apoptotic cell death.

### Induction of a mitochondria-dependent pathway by RSF in 5637 cells

**Figure 3:**
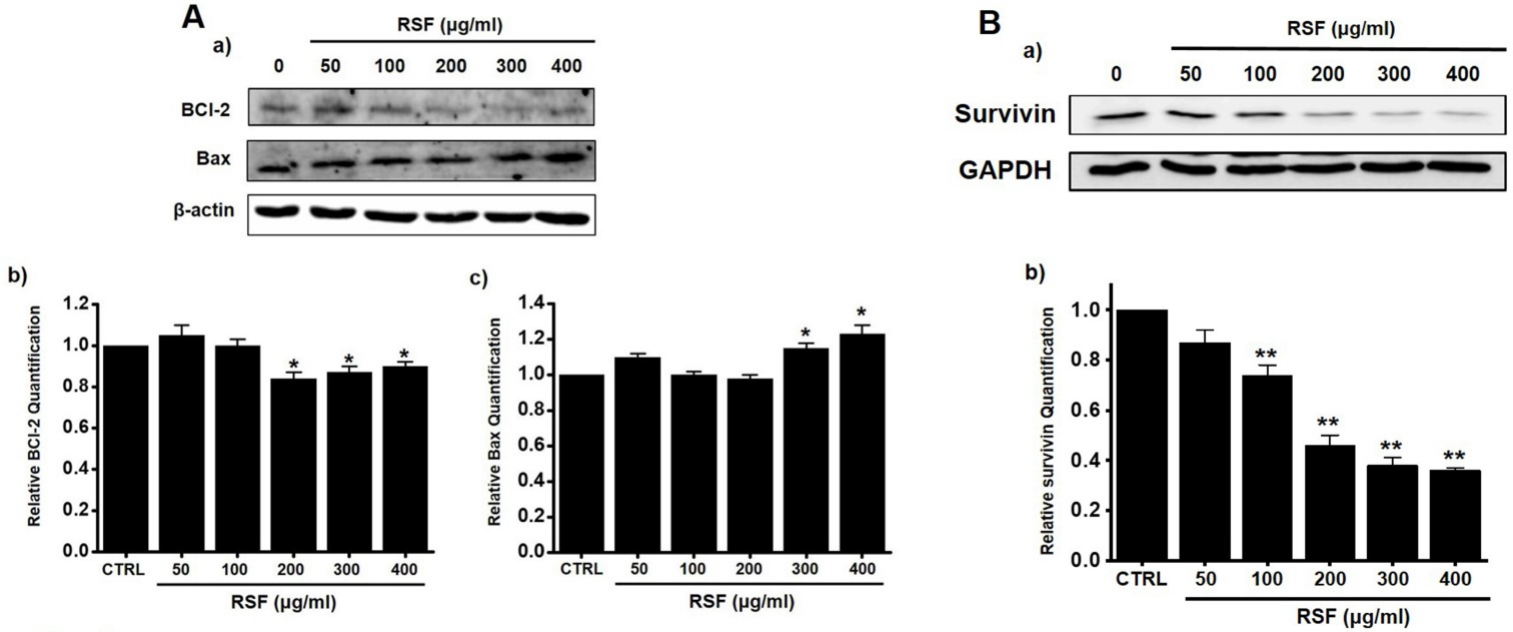
Effects of RSF on BCl-2, Bax and survivin in 5637 cells. (Aa) Western blot was performed on 5637 cells treated with different concentrations of RSF. BCl-2 expression was down-regulated by RSF, whereas Bax expression was up-regulated. (Ab) BCl‑2 and (Ac) Bax protein expressions were normalized versus β‑actin. (Ba) Western blot was performed on 5637 cells treated with different concentrations of RSF and survivin expression was down-regulated by RSF. (Bb) Survivin protein expressions were normalized versus GAPDH. Results are presented as means ± SEMs. *p<0.05. β-Actin was used as the loading control. RSF, ethanolic extract of *Radix Sophorae Flavescentis*; CTRL, control.

Western blotting was used to determine whether RSF-induced apoptosis is regulated by BCl-2 (anti-apoptotic) and Bax (pro-apoptotic). BCl-2 was reduced by RSF, whereas Bax was increased (Fig. 3A). Also, survivin, a member of the family of inhibitor of apoptosis proteins, was reduced by RSF (Fig. 3B). These results indicate RSF-induced apoptosis was dependent on mitochondrial activation in 5637 cells.

### Caspase activations by RSF in 5637 cells

**Figure 4:**
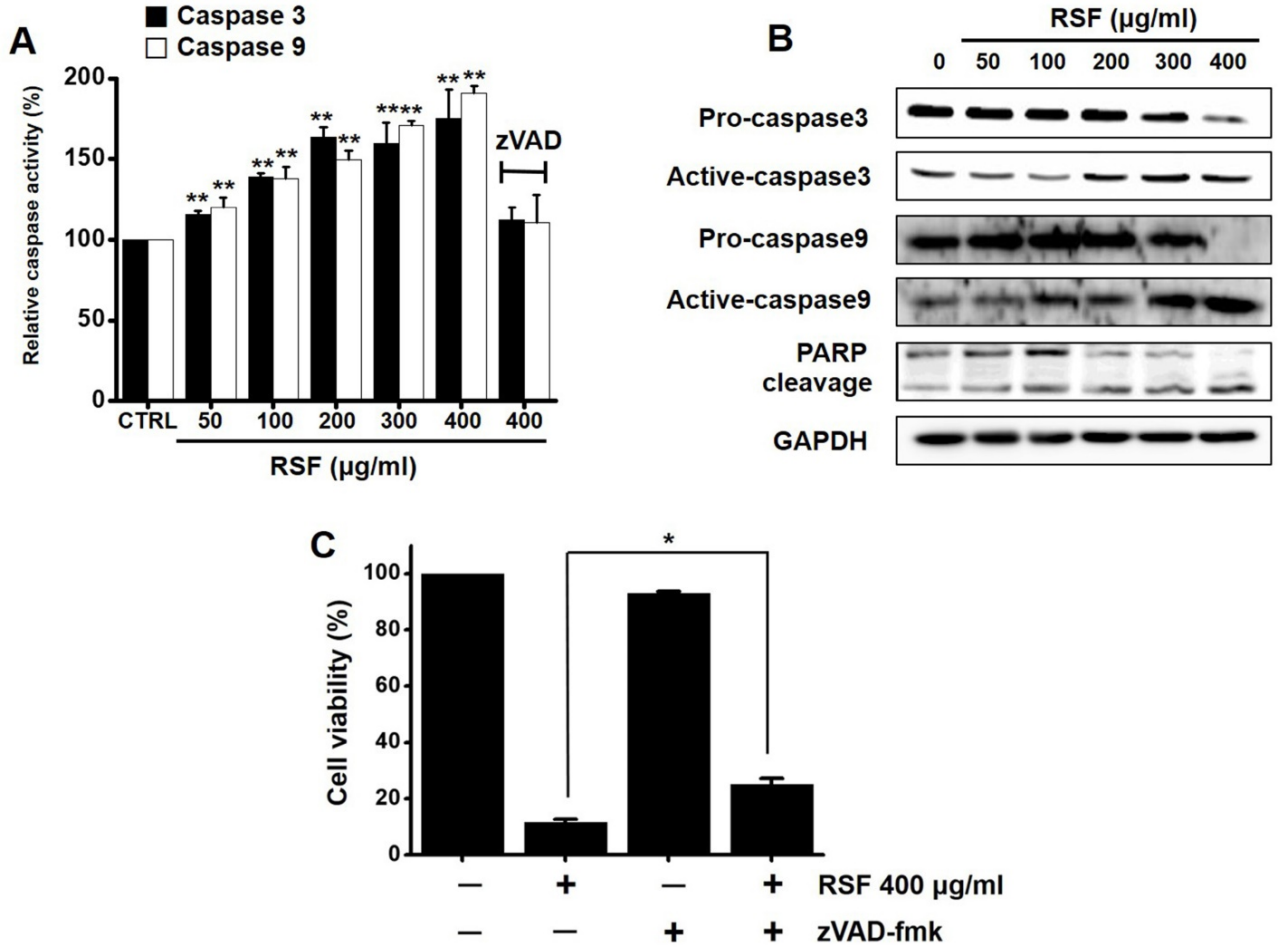
Caspase activations by RSF in 5637 cells*.* (A) RSF dose-dependently increased the activities of caspase -3 and -9. (B) 5637 cells were treated with RSF. Membranes were probed with the indicated antibodies, and GAPDH was used as the internal control. (C) In addition, zVAD-fmk (a pan-caspase inhibitor) pretreatment suppressed RSF-induced reductions in cell viability. Results are presented as means ± SEMs. *p<0.05. **p<0.01. GAPDH was used as the loading control. RSF, ethanolic extract of *Radix Sophorae Flavescentis*; CTRL, control.

Caspases are important mediators of apoptosis via the intrinsic and extrinsic apoptotic pathways. These induce the activation of cytoplasmic endonucleases, which cleaves various substrates, such as poly (ADP‑ribose) polymerase (PARP). Furthermore, the cleavage product of PARP functions as a sign of apoptosis 18. RSF dose-dependently increased the activities of caspase -3 and -9, and zVAD-fmk (a broad-spectrum caspase inhibitor) suppressed these activities (Fig. 4A). Also, western blot showed RSF gradually down-regulated the expressions pro-caspase-3 and -9, gradually up-regulated the active forms of caspase-3 and -9, and up-regulated PARP protein levels (Fig. 4B). When 5637 cells were co-treated with z-VAD-fmk and RSF for 24 h, z-VAD-fmk suppressed RSF-induced apoptosis (Fig. 4C). These results indicate that RSF-induced apoptosis was dependent on caspase activation in 5637 cells.

### Regulations of mitogen-activated protein kinase (MAPK) pathways by RSF in 5637 cells

**Figure 5:**
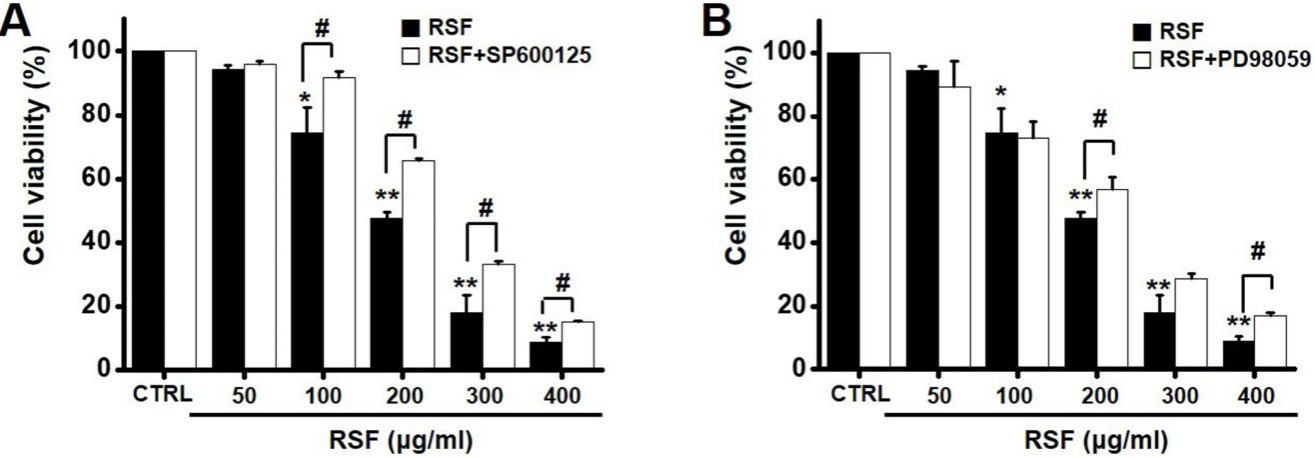
Effect MAPK pathway inhibitors on the effects of RSF in 5637 cells. Cell viabilities were determined using the MTT assay after co-treating cells with RSF plus (A) SP600125 or (B) PD98059. Results are presented as means ± SEMs. *p<0.05. **p<0.01. ^#^*p*<0.05 between treatments. RSF, ethanolic extract of *Radix Sophorae Flavescentis*; CTRL, control.

**Figure 6:**
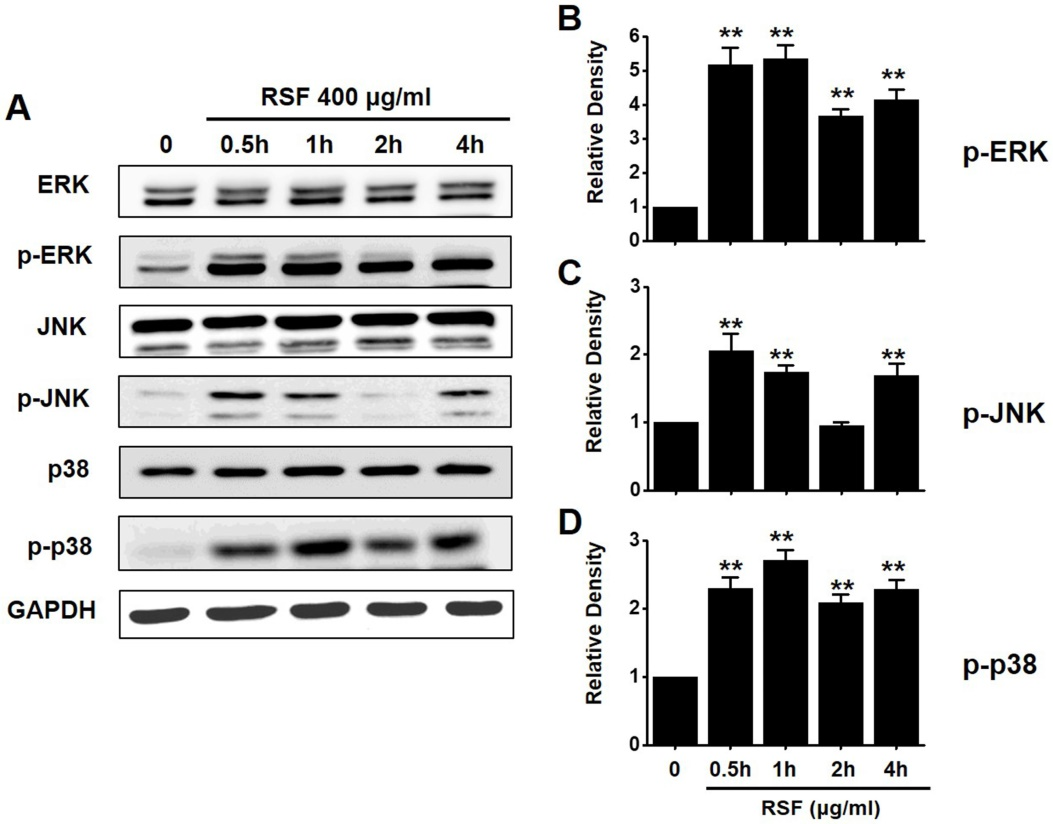
The activations of ERK, JNK, and p38 MAPK pathways in 5637 cells by RSF. 5637 cells were incubated with RSF. (A) Phosphorylations of ERK, JNK, and p38 were confirmed by the Western blot method. (B) Phosphorylated ERK, JNK, and p38 levels are indicated as band densities relative to GAPDH. Results are presented as means ± SEMs. **p<0.01. RSF, ethanolic extract of *Radix Sophorae Flavescentis*; CTRL, control; ERK, extracellular signal regulated kinase; JNK, c‑Jun N‑terminal kinase.

To investigate the effects of RSF on MAPK pathways, cell viabilities were determined after co-treating cells with RSF with or without SP600125 (c-jun NH2-terminal kinase (JNK) II inhibitor) or PD98059 (p42/44 MAPK inhibitor) using the MTT assay. Co-treatment with RSF (50, 100, 200, 300, or 400 µg/ml) and SP600125 inhibited 5637 cell death by 95.9 ± 0.9 %, 91.8 ± 2.0 %, 65.8 ± 0.7 %, 33.4 ± 0.9, and 15.3 ± 0.2 %, respectively (Fig. 5A), and co-treatment with RSF and PD98059 inhibited 5637 cell death by 89.3 ± 7.9 %, 73.0 ± 5.1 %, 56.7 ± 3.9 %, 28.7 ± 1.6 %, and 16.8 ± 1.1 %, respectively (Fig. 5B). To further examine the regulations of these signaling pathways, we investigated RSF-induced phosphorylations of MAPK proteins by Western blot. The phosphorylations of MAPKs, including extracellular signal regulated kinase (ERK), JNK, and p38, increased after treating cells with RSF (400 μg/ml) from 30 min to 4 h (Fig. 6). These results indicate that RSF inhibited the proliferation and induced apoptosis of 5637 cells by modulating MAPK signaling pathways.

### Effect of RSF on intracellular ROS generation in 5637 cells

**Figure 7:**
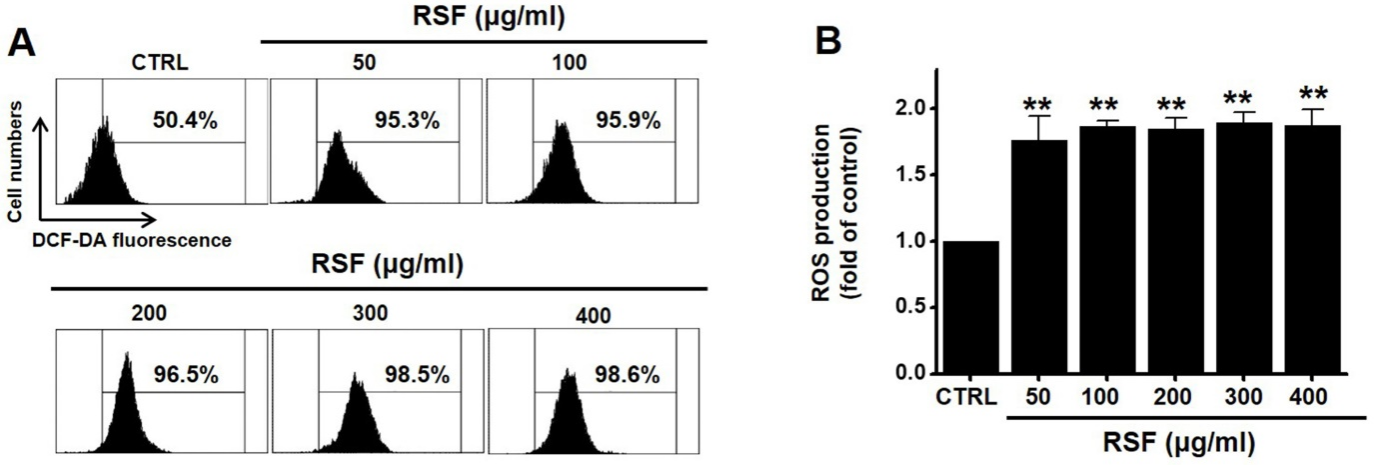
RSF increased ROS levels in 5637 cells. (A) Cells were stained with DCF-DA and then, intracellular ROS levels were measured. (B) Intracellular ROS levels were assessed after treating cells with RSF. ROS levels are expressed as percentages of untreated controls. Results are presented as means ± SEMs. **p<0.001. RSF, ethanolic extract of *Radix Sophorae Flavescentis*; CTRL, control.

Since ROS play a key role in apoptosis, we investigated whether RSF generates ROS in 5637 cells. DCF-DA (a fluorescent dye) was used to check the involvement of ROS in RSF-induced apoptosis. Flow cytometry showed RSF significantly increased ROS levels in 5637 cells (Fig. 7).

## Discussion

Traditional medicines have recently been accepted as useful complementary and alternative treatments for cancer-related diseases 19. *Radix Sophorae Flavescentis* has received much attention because of positive research findings on its anti-cancer efficacy and has also been widely used in traditional medicine for cancer 9-12. Furthermore, the present study demonstrates that an ethanol extract of this herb (RSF) induces the apoptosis of 5637 bladder cancer cells.

Apoptosis plays a crucial role in normal cell development 20. Somewhat surprisingly, although apoptosis is known to play a major role in anticancer therapy, its clinical relevance remains unclear 21. There are two major apoptotic mechanisms: an internal mitochondrial mechanism and an external death receptor mechanism 22. The internal signaling pathway causes apoptosis and mitochondrial signals that react directly with intracellular organelles 23,24. On the other hand, the external signaling pathway initiates apoptosis via receptor-mediated interactions 25. In the present study, BCl-2 and survivin proteins significant decreased when RSF was treated with 5637 cells, while Bax was increased significantly. BCl-2 is an antiapoptotic protein that exhibits oncogenic potential by controlling the intrinsic apoptotic pathway. Our study indicates the decrease in BCl-2 expression in the use of RSF, suggesting that RSF might have the ability to promote apoptosis in 5637 cells. The change in the survivin levels further proves this result. Inhibitor of apoptosis protein (IAP) acts as a control of programmed cell death and apoptosis 26. Survivin is another member of the IAP family of antiapoptotic protein 27. Survivin protein functions to inhibit apoptotic pathway and negatively regulates apoptosis of the cells 27-29. Survivin interacts with caspase-3 and 7, an effector caspases of apoptotic pathway 27-29. In our study, RSF reduces the degree of Survivin and, therefore, RSF may be a powerful inducer of apoptosis in 5637 cells. The proteolytic system, which involves caspases, is a central component of apoptosis 30,31. In the present study, RSF gradually up-regulated the active forms of caspase-3 and -9 and gradually down-regulated the expressions of pro-caspase-3 and -9. In addition, RSF gradually up-regulated the activities of caspase-3 and -9, and these increases were suppressed by zVAD-fmk, which also suppressed RSF-induced cell death. In Fig. 3, the non-treated cells showed PARP cleavage and caspase activation. It is thought that AGS cells themselves are weak and may be caused by cell stress. Also, we think the band may look strong due to the relative difference in density. However, in this experiment, the RSF treatment effect is more obvious than non-treatment, so we think the RSF effect is clearly present. These observations indicate that RSF-induced 5637 cell apoptosis via caspase-dependent death receptor signaling and mitochondria pathways in 5637 cells.

Several ion channels are expressed in a variety of bladder cancer cell lines and have been linked with bladder cell proliferation 32-34. However, the roles played by ion channels in the development and increase of bladder cancer have not been well established. A few reports have shown BK_Ca_ and TREK2 channels regulate 253J cell growth 34,35 and that TRPM8 and TRPV2 are involved in the apoptosis of T24 cells 32,33. However, few have reported that a traditional medicine can cause bladder cancer cell apoptosis by controlling ion channels. Therefore, we suggest additional mechanistic studies be performed to determine whether ion channels are involved in the apoptosis of bladder cancer cells by RSF.

MAPKs are involved in intracellular action mechanisms during proliferation, differentiation, stress responses, and apoptosis 36. Furthermore, MAPK pathways, such as those of ERK1/2, p38 MAPK, and JNK, have been implicated in chemotherapy and particularly in drug toxicity 36,37. In the present study, we found that RSF activated the ERK, p38, and JNK pathways and reduced 5637 cell survival. In addition, the inhibition of MAPK signaling by PD98059 or SP600125 protected 5637 cells from RSF induced cell death, which suggests the activation of MAPK cascades acts to prevent the proliferation of 5637 cells.

In conclusion, the present study shows RSF inhibits 5637 cell growth and increases the proportion of cells in the sub‑G1 phase. In addition, RSF‑induced apoptosis was found to be associated with the down-regulation of BCl‑2 or survivin and the up-regulation of Bax. Furthermore, RSF activated caspase‑3 and -9 and MAPK cascades and increased intracellular ROS levels. We hope that this research provides an opportunity to develop a cure for bladder cancer.

## References

[1] Richters A, Aben KKH, Kiemeney LALM (2019). The Global Burden of Urinary Bladder Cancer: An Update. World J Urol.

[2] Xia Y, Yuan M, Li S (2018). Apigenin Suppresses the IL-1β-Induced Expression of the Urokinase-Type Plasminogen Activator Receptor by Inhibiting MAPK-Mediated AP-1 and NF-κB Signaling in Human Bladder Cancer T24 Cells. J Agric Food Chem.

[3] Martyn-Hemphill C, Mak D, Khan MS (2013). Recent advances in diagnosis and treatment of transitional cell carcinoma of the bladder. Int J Surg.

[4] Witjes JA, Kolli PS (2008). Apaziquone for non-muscle invasive bladder cancer: a critical review. Expert Opin Investig Drugs.

[5] Zhang S, Ding D, Zhang X (2014). Maslinic acid induced apoptosis in bladder cancer cells through activating p38 MAPK signaling pathway. Mol Cell Biochem.

[6] Lee H, Kim W, Kang HG (2020). Geranium thunbergii extract-induced G1 phase cell cycle arrest and apoptosis in gastric cancer cells. Anim Cells Syst.

[7] Li X, Yang G, Li X (2013). Traditional Chinese medicine in cancer care: A review of controlled clinical studies published in Chinese. PLoS One.

[8] Liu J, Zhu M, Shi R (2003). Radix Sophorae flavescentis for chronic hepatitis B: A systematic review of randomized trials. Am J Chin Med.

[9] Wang Y, Han C, Fang X (2013). Effect of kushen (Radix Sophorae Flavescentis) extract on laryngeal neoplasm Hep2 cells. J Tradit Chin Med.

[10] Yang X, Cai W, Yang Q (2015). Compound Radix Sophorae Flavescentis exerts antitumor effects by inhibiting the proliferation and inducing the apoptosis of esophageal carcinoma TE-8 cells. Oncol Lett.

[11] Wang Q, Xu J, Li X (2017). Comprehensive two-dimensional PC-3 prostate cancer cell membrane chromatography for screening anti-tumor components from Radix Sophorae Flavescentis. J Sep Sci.

[12] Kim JS, Shin SJ, Kim JN (2019). Radix Sophorae Flavescentis inhibits proliferation and induces apoptosis of AGS human gastric cancer cells. Mol Med Rep.

[13] Sarfaraz S, Adhami VM, Syed DN (2008). Cannabinoids for cancer treatment: progress and promise. Cancer Res.

[14] Wong RS (2011). Apoptosis in cancer: from pathogenesis to treatment. J Exp Clin Cancer Res.

[15] Lu ML, Xiang XH, Xia SH (2016). Potential signaling pathways involved in the clinical application of oxymatrine. Phytother Res.

[16] Huang J, Xu H (2016). Matrine: Bioactivities and structural modifications. Curr Top Med Chem.

[17] Luo KW, Lung WY, Xie C (2018). EGCG Inhibited Bladder Cancer T24 and 5637 Cell Proliferation and Migration via PI3K/AKT Pathway. Oncotarget.

[18] Boulares AH, Zoltoski AJ, Contreras FJ (2002). Regulation of DNAS1L3 endonuclease activity by poly(ADP-ribosyl)ation during etoposide-induced apoptosis. Role of poly(ADP-ribose) polymerase-1 cleavage in endonuclease activation. J Biol Chem.

[19] Yin SY, Wei WC, Jian FY (2013). Therapeutic applications of herbal medicines for cancer patients. Evid Based Complement Alternat Med. 2013; 302426.

[20] Schultz DR, Harrington WJ Jr (2003). Apoptosis: programmed cell death at a molecular level. Semin Arthritis Rheum.

[21] Kim R, Emi M, Tanabe K (2006). The role of apoptosis in cancer cell survival and therapeutic outcome. Cancer Biol Ther.

[22] Igney FH, Krammer PH (2002). Death and anti-death: tumour resistance to apoptosis. Nat Rev Cancer.

[23] Okada H, Mak TW (2004). Pathways of apoptotic and non-apoptotic death in tumour cells. Nat Rev Cancer.

[24] Jin Z, El-Deiry WS (2005). Overview of cell death signaling pathways. Cancer Biol Ther.

[25] Kroemer G, Reed JC (2000). Mitochondrial control of cell death. Nat Med.

[26] Schimmer AD (2004). Inhibitor of Apoptosis Proteins: Translating Basic Knowledge Into Clinical Practice. Cancer Res.

[27] Mobahat M, Narendran A, Riabowol K (2014). Survivin as a preferential target for cancer therapy. Int J Mol Sci.

[28] Chen X, Duan N, Zhang C (2016). Survivin and tumorigenesis: molecular mechanisms and therapeutic strategies. J Cancer.

[29] Tang C, Lu YH, Xie JH (2009). Downregulation of survivin and activation of caspase-3through the PI3K/Akt pathway in ursolic acid-induced HepG2 cell apoptosis. Anticancer Drugs.

[30] Doustvandi MA, Mohammadnejad F, Mansoori B (2017). The interaction between the light source dose and caspase-dependent and -independent apoptosis in human SK-MEL-3 skin cancer cells following photodynamic therapy with zinc phthalocyanine: A comparative study. J Photochem Photobiol B.

[31] Xue M, Ji X, Xue C (2017). Caspase-dependent and caspase-independent induction of apoptosis in breast cancer by fucoidan via the PI3K/AKT/GSK3β pathway in vivo and in vitro. Biomed Pharmacother.

[32] Li Q, Wang X, Yang Z (2009). Menthol induces cell death via the TRPM8 channel in the human bladder cancer cell line T24. Oncology.

[33] Yamada T, Ueda T, Shibata Y (2010). TRPV2 activation induces apoptotic cell death in human T24 bladder cancer cells: a potential therapeutic target for bladder cancer. Urology.

[34] Park KS, Han MH, Jang HK (2013). The TREK2 Channel Is Involved in the Proliferation of 253J Cell, a Human Bladder Carcinoma Cell. Korean J Physiol Pharmacol.

[35] Kim Y, Kim WJ, Cha EJ (2011). Quercetin-induced growth inhibition in human bladder cancer cells is associated with an increase in Ca2+-activated K+ channels. Korean J Physiol Pharmacol.

[36] Chang L, Karin M (2001). Mammalian MAP kinase signalling cascades. Nature.

[37] Taylor CA, Zheng Q, Liu Z (2013). Role of p38 and JNK MAPK signaling pathways and tumor suppressor p53 on induction of apoptosis in response to Ad-eIF5A1 in A549 lung cancer cells. Mol Cancer.

